# Supplementary material for: Comprehensive Annotation and Functional Exploration of MicroRNAs in Lettuce
Source: Front Plant Sci. 2021 Dec 24;12:781836. doi: 10.3389/fpls.2021.781836 (PMC8739914; doi:10.3389/fpls.2021.781836)
Supplement: Supplementary file 3 [file Data_Sheet_9.ZIP › GSM2230751.plot/Lsa-miRN1690_Lsat_1_v5_gn_4_148421.1_1106_TPlot.pdf]

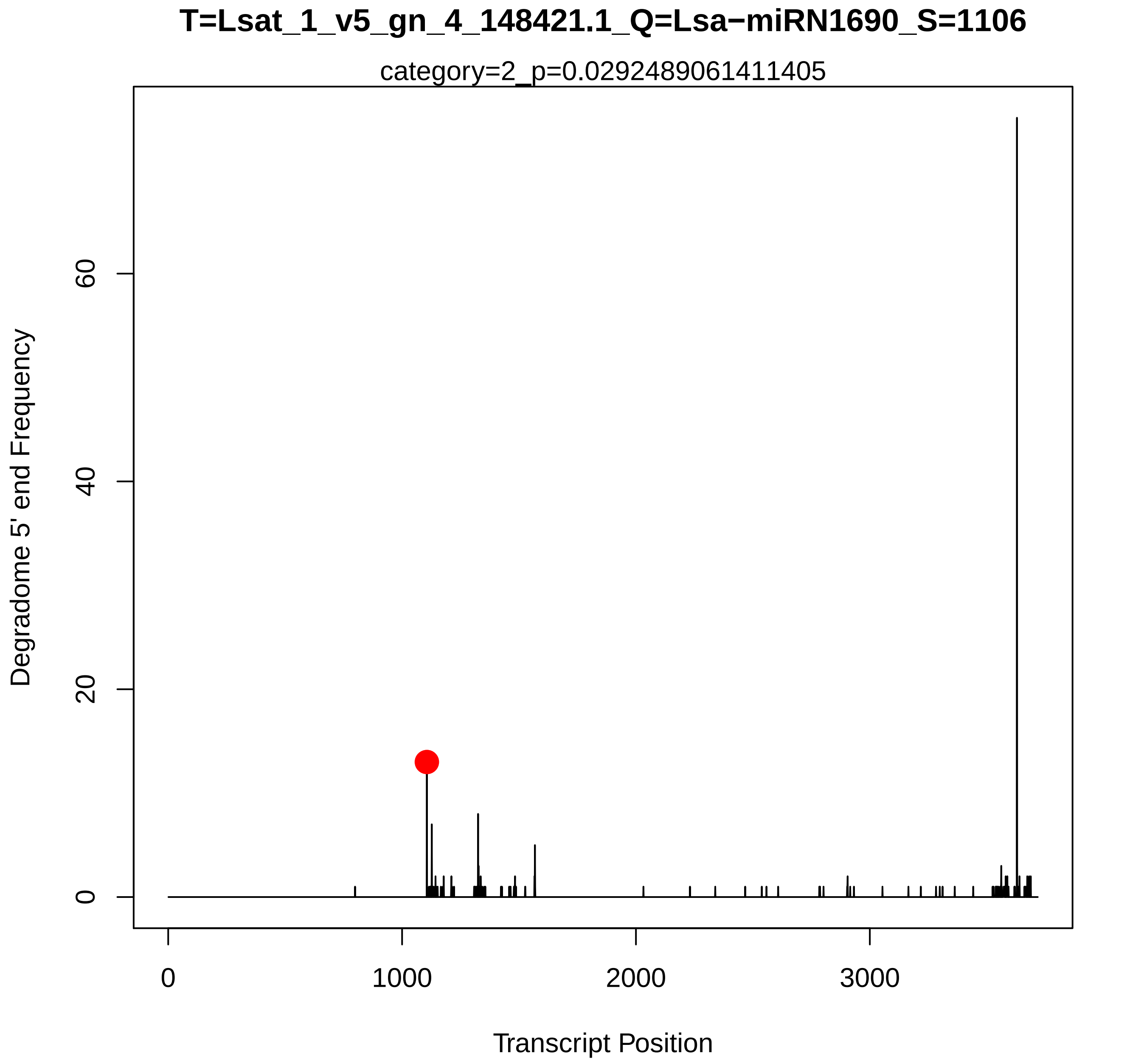
T=Lsat_1_v5_gn_4_148421.1_Q=Lsa−miRN1690_S=1106
category=2_p=0.0292489061411405
Degradome 5' end Frequency
0
20
40
60
0
1000
2000
3000
Transcript Position
